# The transcriptome of a “sleeping” invader: *de novo *assembly and annotation of the transcriptome of aestivating *Cornu aspersum*

**DOI:** 10.1186/s12864-017-3885-1

**Published:** 2017-06-28

**Authors:** Aristeidis Parmakelis, Panayiota Kotsakiozi, Christos K. Kontos, Panagiotis G. Adamopoulos, Andreas Scorilas

**Affiliations:** 1 Department of Ecology and Taxonomy, Faculty of Biology, National and Kapodistrian University of Athens, Athens, Greece; 2 Department of Biochemistry and Molecular Biology, Faculty of Biology, National and Kapodistrian University of Athens, Athens, Greece

**Keywords:** Land-snail, Next-generation sequencing (NGS), Non-model organism, RNA-seq

## Abstract

**Background:**

*Cornu aspersum* is a quite intriguing species from the point of view of ecology and evolution and its potential use in medical and environmental applications. It is a species of economic importance since it is farmed and used for culinary purposes. However, the genomic tools that would allow a thorough insight into the ecology, evolution, nutritional and medical properties of this highly adaptable organism, are missing. In this work, using next-generation sequencing (NGS) techniques we assessed a significant portion of the transcriptome of this non-model organism.

**Results:**

Out of the 9445 *de novo *assembled contigs, 2886 (30.6%) returned significant hits and for 2261 (24%) of them Gene Ontology (GO) terms associated to the hits were retrieved. A high percentage of the contigs (69.4%) produced no BLASTx hits. The GO terms were grouped to reflect biological processes, molecular functions and cellular components. Certain GO terms were dominant in all groups. After scanning the assembled transcriptome for microsatellites (simple sequence repeats, SSRs), a total of 563 SSRs were recovered. Among the identified SSRs, trinucleotide repeats were the predominant followed by tetranucleotide and dinucleotide repeats.

**Conclusion:**

The annotation success of the transcriptome of *C. aspersum* was relatively low. This is probably due to the very limited number of annotated reference genomes existing for mollusc species, especially terrestrial ones. Several biological processes being active in the aestivating species were revealed through the association of the transcripts to enzymes relating to the pathways. The genomic tools provided herein will eventually aid in the study of the global genomic diversity of the species and the investigation of aspects of the ecology, evolution, behavior, nutritional and medical properties of this highly adaptable organism.

## Background

The pulmonate species *Cornu aspersum* (Müller, 1774) is one of the core species of the Mediterranean malacofauna [1], especially of the insular ecosystems such as those of the Aegean Sea [2–4]. A typical inhabitant of the Mediterranean, this species succeeded to expand its distribution and is currently present in climatic regions deviating significantly from its favorable climate, that of the Mediterranean. Presently the species occupies areas in the American continent, in Australia and Asia. The dispersal of *C. aspersum* was intentionally or stochastically facilitated by human activities and it is estimated to have begun at some point during the Neolithic period, approximately 8500 years before present [5]. The different climatic conditions prevailing in the areas *C. aspersum* is presently occupying, are indicative of a tremendous adaptive capability of the species in terms of biological, ecological and physiological responses. Besides the climatic differences, *C. aspersum* was also faced with competition from the native gastropod species. Considering that the species usually exhibits quite dense populations, it can be considered a very successful colonizer. In several areas, the species is even posing as an important crop pest [5, 6]. Besides the impressive colonizing ability of *C. aspersum*, another important aspect is the multiple ways that this organism is being exploited by humans. It is considered a special delicacy in culinary, and it is being intensively farmed. At the same time, some of its excretions are being investigated for potential medical applications [7, 8], whereas they have already been used for healing burn-wounds [8]. Additionally, it is used as an index of soil and air quality in urban and industrial areas [9–12], whereas products originating from closely related species such as *Helix pomatia* or *Helix lucorum* are used in cancer diagnostic protocols [13–17].

Considering all the above, *C. aspersum* is a quite intriguing species from the point of view of ecology and evolution and its potential use in medical and environmental applications. At the same time, it is a species with an already acknowledged economic importance as a farmed species used for culinary purposes. However, the genomic tools that would allow a thorough insight into the ecology, evolution, nutritional and medical properties of this highly adaptable organism, are missing.

In this work, we applied next-generation sequencing (NGS) techniques to assess the transcriptome of this non-model organism. More specifically, we implemented an RNAseq analysis that is an increasingly popular technique for genome-wide ecological transcriptomics [18]. It uses NGS methods to characterize RNA transcripts using high-throughput sequencing of a cDNA library to generate hundreds of thousands of DNA fragments [19]. An advantage over other available NGS approaches is that RNA-seq data is directly derived from functional genomic elements, mostly protein-coding genes [19]. Thus, transcriptome sequence constitutes a meaningful resource to develop a large number of popular molecular markers such as single-nucleotide polymorphisms (SNPs) and microsatellites (simple sequence repeats, SSRs). In our approach, we used the Ion Torrent™ platform to sequence a portion of the transcriptome of the organism during the species’ dormant state. We conducted a *de* *novo* assembly of the transcriptome and used bioinformatics tools to annotate, assign functional attributes to the assembled sequences and screen the transcriptome for the presence of SSRs. Our ultimate goal was to provide genomic tools that will eventually aid in the study of the global genomic diversity of the species and investigate aspects of the ecology, evolution, behavior, nutritional and medical properties of this highly adaptable organism. In addition, issues relating to the exploited dispersal corridors, origin of invading populations and genes offering adaptive advantages, could probably be addressed much more effectively through the use of novel genomics tools that will become available based on the generated transcriptome data.

## Results

### Ion semiconductor sequencing and de novo transcriptome assembly

**Fig. 1:**
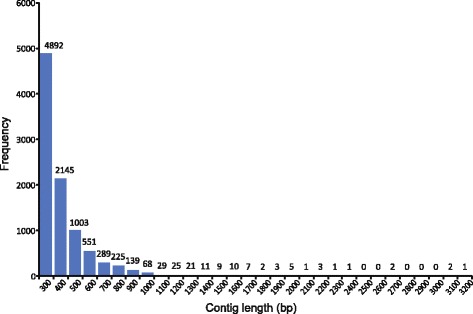
Size distribution of the *de novo *assembled contigs from the transcriptome of the foot muscle of aestivating *C. aspersum*

The Ion Torrent PGM™ platform generated 73,853,613 nucleotide data in a total of 669,345 raw reads with a GC content of 41.94%. After filtering the low-quality reads with the FASTQ-quality-filter tool, and following the removal of the mitochondrial 16S ribosomal RNA (rRNA) fragments, we obtained 527,273 high quality reads. These served as the input for the short-read assembler Trinity. The Trinity software generated 9445 contigs. Based on all the contigs generated, the average and median lengths of the contigs were 360 bp and 295 bp, respectively. The minimum length of a contig was 201 bp and the maximum was 3101 bp. A percentage of 14.9% of the contigs had sizes between 500 and 1000 bp, whereas 1.42% of the contigs exceeded 1000 bp in length. The size distribution of the generated contigs is presented in Fig. 1. The N50 (50% of total assembled sequences having this length or longer contigs) for all the contigs considered was 365 bp. A typical Trinity transcriptome assembly will have the vast majority of all reads mapping back to the assembly. The RNA-seq read representation analysis performed with Trinity, and using the total number of contigs, estimated that 19.19% (101,160 reads) of the initial raw reads (527,273), could be aligned once back to the assembly, whereas 46.40% (244,649 reads) could be aligned more than once. In total, 65.58% (345,809 reads) of the total raw reads could be mapped back to the assembly.

### Functional annotation of contigs

**Fig. 2:**
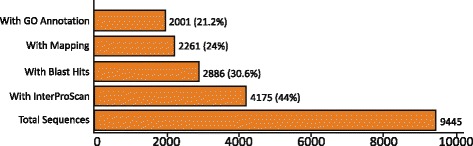
Summary of the results of the automated annotation of the *de novo*assembled contigs using the Blast2GO v.4.0.7 software

**Fig. 3:**
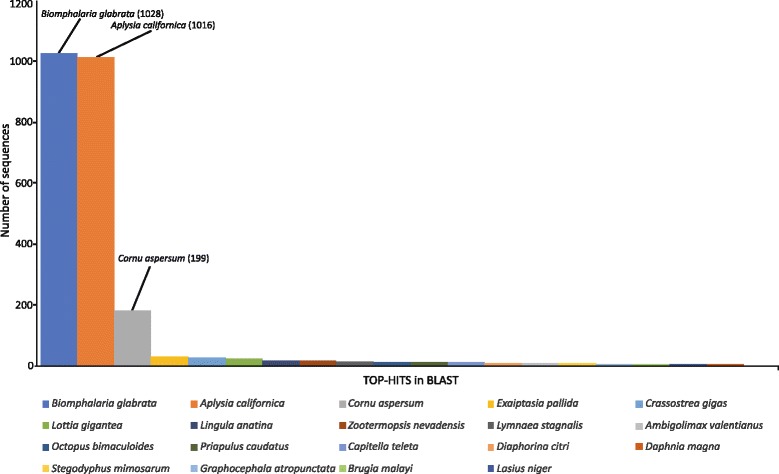
The species most commonly represented in the BLAST hits of the *de novo*assembled contigs

**Fig. 4:**
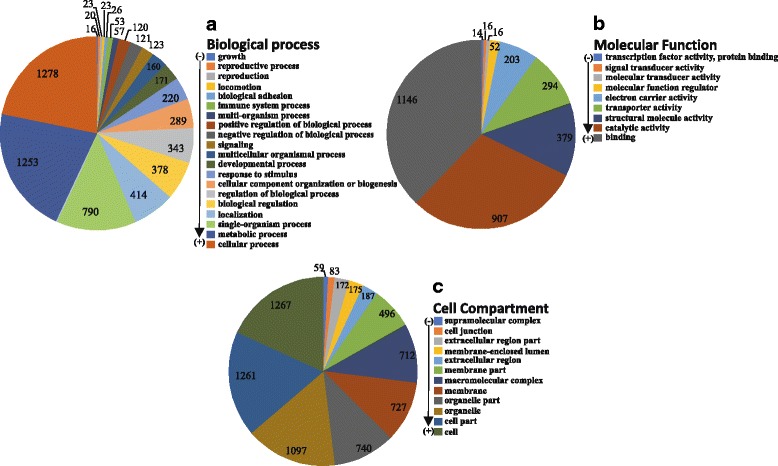
The GO terms relating to (**a**) biological process, (**b**) molecular function and (**c**) cellular component categories assigned to the contigs of the *de novo*assembly of *C. aspersum* transcriptome. The arrow and the (−) and (+) signs on the left of each pie-chart legend, indicate the direction of increase in the number of contigs corresponding to the biological process, molecular function or cell compartment denoted in the respective pie-chart legend

Out of the 9445 contigs that were queried against the NR database, 2886 (30.6%) returned significant hits and for 2261 (24%) of them Gene Ontology (GO) terms associated to the hits were retrieved (mapping). A high percentage of the contigs (69.4%) produced no BLASTx hits, whereas 4175 (44%) contigs generated InterPro identity results. A diagram summarizing the above is presented in Fig. 2. More than 70% of the BLAST hits were made up of different mollusk species. The species with the highest number of hits were the freshwater species *Biomphalaria glabrata* (33%) and the marine species *Aplysia californica* (32.6%). The NR database included several sequences originating from the species *C. aspersum* investigated herein, and these provided a BLASTx hit up to 5.8% (Fig. 3). Other marine, terrestrial and freshwater molluscs (i.e. *Ambigolimax valentinus, Crassostrea gigas, Lottia gigantea, Lymnaea stagnalis*) were also included in the hits but accounted for much lower portions of the overall hits. The annotations of all the contigs under the GO terms assigned at level 2 are shown in Fig. 4. The GO terms were grouped to reflect biological processes, molecular functions and cellular components. It is evident that in all groups, there were certain GO terms that were dominant.

### SSRs detection in the assembled contigs

**Fig. 5:**
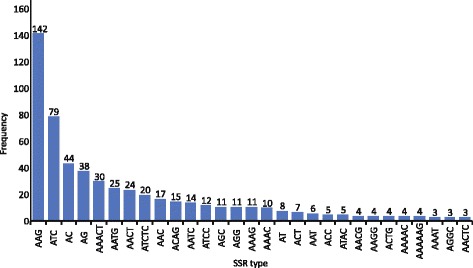
The different SSR types discovered in the assembled contigs and the frequency of their appearance within the assembled transcriptome

For SSR identification, we examined the 9445 contigs and a total of 563 SSRs were found. Among the identified SSRs, trinucleotide repeats (278, 49.4%) were the predominant followed by tetranucleotide (134, 23.8%) and dinucleotide (90, 16.0%) repeats. Out of the trinucleotide repeats the type AAG/CTT (25.2%) and ATC/GAT (14.0%) were the prevailing ones. In the tetranucleotides AATG/CATT (4.4%) and AACT/AGTT (4.3%) exhibited higher frequencies. In the dinucleotides, the type AC/GA (7.8%) was slightly more frequent than AG/CT (6.7%). The frequency of each SSR type obtained in this study is shown in Fig. 5.

## Discussion

Precise and accurate *de novo *assembly and annotation of transcriptomes, has been acknowledged as a critically important step for assemblies generated from short reads (~100-150 bp), especially when working with non-model organisms that lack a reference genome [20–22]. Evaluating the performance of different short read assemblers in such *de novo *transcriptome assembly efforts, is one way to increase the degree of confidence in the generated outcome. Regarding, gastropods’ transcriptomes and the Ion Torrent PGM™ system, the performance of different short read assemblers has already been assessed [20]. In this study, we targeted the transcriptome of *C. aspersum*. Using the Ion Torrent PGM™ system, and after quality filtering, 527,273 high quality reads were generated. The short read assembler used to assemble the raw reads, was the Trinity software that was found to outperform other available ones [20]. The Trinity assembler generated 9445 contigs with lengths ranging from 201 bp up to 3101 bp (Fig. 1). The quality of the assembly was high as corroborated by the quality assessment metrics estimated. The N50 length of 365 bp and the average contig length of 360 bp are comparable to other transcriptome sequencing studies of molluscan species [20, 21, 23]. However, there is a very big difference between the amount of sequence data and number of contigs generated in our study and those reported in a study involving the species *C. aspersum* from Australia and the Illumina HiSeq™ 2000 system [24]. In the latter study, the output of the sequencing reads was 21,273,910, whereas Trinity generated 160,490 contigs. The differences between the sequence data generated in our study, and those of [24] are most likely due to the different sequencing output efficiency of the NGS platforms. This issue has been pointed before in the comparison of the transcriptome data generated for *Nerita melanotragus* [20] and *Lymnaea stagnalis* [25] using the Ion Torrent PGM™ and an Illumina® platform, respectively. Additional factors that could account for the differences between our study and that of [24], are: a) the fact that we have used only the foot muscle for total RNA extraction in contrast to the whole animal being used in [24], and b) the specimen we used was aestivating, a condition under which the majority of the biological processes are either suppressed or not active at all, and thus transcription activity is significantly reduced.

Out of the 9445 contigs assembled only a small fraction of 30.6% returned significant BLASTx hits (Fig. 2). The low level of annotation success is largely due to the very limited number of annotated reference genomes existing for mollusc species, especially terrestrial ones. So far, 23 transcriptomes of terrestrial (non-freshwater) gastropods have been generated [23, 24, 26], but none has yet been comprehensively annotated. Thus, for terrestrial gastropods, annotation still relies on the reference genomes of phylogenetically distant freshwater and marine species. More likely in a transcriptome study involving a terrestrial gastropod, the species with the top blast hits will be the species *Aplysia californica* and *Biomphalaria glabrata* for which extensive genomic information is available in public databases. This is the case in our study, with the majority of the blast hits being assigned to *B. glabrata* and *A. californica* (Fig. 3)*.* The very limited *C. aspersum* sequences being present in the public databases, were the next successful group of sequences for which significant number of hits were obtained.

The 9445 contigs assembled from the RNA extracted from the foot muscle of an aestivating *C. aspersum* individual, were referenced to the NR database at NCBI as BLASTx queries. Following that, GO terms were assigned to them, and the biological processes and the molecular functions that are likely to be active in the aestivating individual are presented in Fig. 4. In addition, there were several contigs that were not assigned a specific GO name, but exhibited matches to InterPro identities. The GO terms that dominated the biological process category were cellular process, metabolic process, single-organism process, localization and biological regulation. Several contigs were also found to be involved in processes such as cellular component organization or biogenesis and response to stimulus. In the category of molecular function, the most prominent GO terms included binding, catalytic activity, structural molecule activity, transporter activity and electron carrier activity. In the cellular compartment category, the highest proportions of annotations were shown by cell, cell part, organelle, organelle part, membrane and macromolecular complex. The specimen used in this study was aestivating, and as a result it was in a state in which all the biological processes were reduced to a minimum level. Even so, the biological processes and molecular functions exhibit a pattern that is comparable to that of active individuals. For instance, in the recent study of the terrestrial snail *Koreanohadra kurodana* [23], whose messenger RNA (mRNA) was collected during the active state of the specimens, the transcripts being dominant in the assemblies, relate to the same biological processes and molecular functions, that were prevailing in the case of the mRNA harvested from the foot muscle of the aestivating *C. aspersum* of the present study. To our understanding, this is an indication that the physiological differences between an aestivating and an active terrestrial gastropod, cannot be reflected in the broader biological processes/molecular functions being tracked in these cases. Identifying the transcripts and genes that are involved in the fine tuning of an aestivating individuals’ physiological pathways, will require the comparative analyses of the transcriptomes of active and aestivating individuals. Such an experimental design through a differential expression analysis of the transcriptomes, could provide some insights into already known genes having a role in regulating these processes, or disclose new transcripts and genes relating to these processes. In addition, since during aestivation the terrestrial gastropods are faced with adverse environmental conditions, such a comparative analysis could also reveal those known or novel transcripts that are involved in the adaptation of species to harsh or unfavourable environmental conditions, and thus enable a species to increase or maintain its distributional range.

An important utility of high-throughput transcriptome characterization is to identify SSR and SNP markers within the transcribed regions. Both types of markers contain adequate levels of information to assess population diversity at the genetic level and can be used to infer the fine spatial scale phylogeography of the species under question. There were several limitations in the development of these type of markers in non-model organisms. The limitations were mainly time and cost, since the markers had to be screened from genomic libraries. Nowadays, screening of whole genomes is feasible through the development of appropriate bioinformatics tools [27] applied on NGS derived genomes. In this study, we screened the transcriptome for SSRs and identified 563 different SSR markers. The SSRs recovered ranged from di- to hexanucleotide type of repeats. The markers identified have to be tested regarding their polymorphism [28] before being added to the arsenal of the markers already available [29, 30] for the species. The additional markers identified in this study will permit a finer spatial scale of the study of the species’ phylogeographic history and global population structure.

## Conclusions

In this study, we report the first transcriptome originating from the foot muscle of an aestivating *C. aspersum* individual. The annotation success was relatively low (30.6%). This is due to the very limited number of annotated reference genomes existing for mollusc species, especially terrestrial ones. The 9445 contigs were classified into biological and molecular processes as well as into cellular component categories. Several biochemical pathways being active in the aestivating species were revealed through the association of the transcripts to enzymes relating to the pathways. After scanning the transcriptome for SSRs, 563 SSR markers were recovered. The genomic tools provided herein will eventually aid in the study of the global genomic diversity of the species and the investigation of aspects of the ecology, evolution, behavior, nutritional and medical properties of this highly adaptable organism.

## Methods

### Sampling of snails

Several *Cornu aspersum* (Müller, 1774) individuals were collected from the region of Petra in NW Lesvos island (Greece). The sampling took place in August 2015. The sampled individuals were aestivating at the time of collection. The aestivating state of the specimens, was indicated both by the lack of activity and by the formation of an epiphragm covering the shell’s opening. The formation of the epiphragm is a strong indication of a land-snail having entered a dormancy state [31]. Two days after the collection of the specimens from the field, the sampled individuals were placed at −80 °C and stored there until RNA extraction.

### NGS library construction, template preparation, and semi-conductor sequencing

The foot muscle of a single aestivating specimen was homogenized and then dissolved in TRI® Reagent Solution (Ambion™, ThermoFisher Scientific, Carlsbad, CA, USA). Among the different body parts of a land-snail, we selected the foot muscle to be harvested for RNA. The foot muscle together with the hepatopancreas, metabolize and store lipids, thus are physiologically quite active organs during periods of adverse conditions such as the aestivation period [31, 32]. At the same time, numerous genes relating to the epiphragm that seals off the aperture during hibernation and aestivation, are encoded in the foot muscle [33]. Aiming at obtaining as much transcriptomic information as possible relating to the aestivating status of our specimen, the foot muscle seemed as the most reasonable choice. Total RNA was isolated from the pulverized tissue, and its purity and concentration were assessed spectrophotometrically using the BioSpec-nano Micro-volume UV-Vis Spectrophotometer (Shimadzu Scientific Instruments, Columbia, MD, USA). After having checked the quality and integrity of the extracted RNA using agarose gel electrophoresis, high purity polyadenylated (poly(A)) RNA was isolated by implementing the Dynabeads® mRNA DIRECT™ Micro Purification Kit (Ambion™). Next, we used the Ion Total RNA-Seq Kit v2 (Ion Torrent™, ThermoFisher Scientific, Carlsbad, CA, USA) and the Ion Xpress™ RNA-Seq Barcode 1–16 Kit (Ion Torrent™) to prepare a representative cDNA library for strand-specific RNA sequencing. The amplified library served for sequencing template preparation using the Ion PGM™ Template OT2 200 Kit (Ion Torrent™) on an Ion OneTouch™ 2 Instrument (Ion Torrent™). The enriched template was loaded on an Ion 318™ Chip v2 (Ion Torrent™) and sequenced on the Ion Personal Genome Machine® (PGM™) system (Ion Torrent™), using the Ion PGM™ Sequencing 200 Kit v2 (Ion Torrent™). All steps were performed according to the manufacturer’s guidelines. Finally, sequencing data were obtained using the Ion PGM™ Torrent Server and Torrent Suite™ Software (Ion Torrent™).

### *de novo*assembly and transcriptome analysis

The raw sequencing reads were converted to FASTQ files by the Torrent Suite Software. The reads in the FASTQ files were trimmed at their 3′ ends of regions of trailing low quality and adapter sequences were clipped. Additional steps of raw reads quality assessment was performed using the FASTQ-quality-filter tool of the FastX-Toolkit [34]. Reads were maintained based on a quality threshold of Q > 10 and the minimum percent of bases retaining this quality level was set to 90%. Following the quality filtering, reads were evaluated with FASTQC (available from http://www.bioinformatics.babraham.ac.uk/projects/fastqc/). A set of overrepresented sequences were identified by FASTQC and these corresponded to the mitochondrial 16S rRNA fragments. As it is mentioned in [35], failure to remove all rRNA sequences can lead to misclassifications and erroneous conclusions during the downstream analysis. In addition, it is estimated that misannotations of rRNA as proteins may cause up to 90% false positive matches of rRNA-like sequences in meta-transcriptomic studies [36]. Therefore, the 16S rRNA fragments were removed from the dataset using the SortMeRNA tool [37]. All downstream analyses were performed based on the filtered and 16S rRNA free dataset.

The high-quality reads were assembled into contiguous sequences (contigs) using the short read de novo assembler Trinity (version 2.3.2 [38]). Trinity was run under the default parameters. The Trinity *de novo *program has been used extensively as an assembly algorithm. The program compiles the raw sequence data into a number of de Bruijin graphs and ultimately reports transcripts (contigs) in their final form. Following contig generation, the transcriptome assembly was assessed for quality following some of the suggestions provided in the Trinityrnaseq wiki page. Specifically, we: i) examined the RNA-seq read representation, and ii) computed the Nx statistics. Additionally, we calculated standard metrics of quality including number of contigs and average and median contig length.

After the quality control of the transcriptome assembly, the contigs were referenced to the NR protein database at NCBI as BLASTx queries using the Blast2GO v.4.0.7 software [39]. During the BLASTx searches the threshold was set to an e-value of 1 × 10^−5^. The InterProScan software [40] as implemented in Blast2GO v.4.0.7 was used to perform protein domain analysis and scan the contigs against the InterPro resource [41]. GO terms were assigned using the Blast2GO v.4.0.7 software. The GO annotation analyses were performed at level 2.

### Discovery of SSR markers

All the Trinity generated contigs were screened for the presence of SSR markers using the SciRoKo software [27]. The SSR search mode was set to perfect: total length, with minimum total length set to 15 and the minimum repeats set to 3. All other parameters were left at their default values. Mononucleotides were not considered at all.

## Abbreviations

GO: Gene ontology
mRNA: Messenger RNA
NGS: Next-generation sequencing
rRNA: Ribosomal RNA
SNP: Single-nucleotide polymorphism
SSRs: Simple sequence repeats
